# miRNA–mRNA network regulation in the skeletal muscle fiber phenotype of chickens revealed by integrated analysis of miRNAome and transcriptome

**DOI:** 10.1038/s41598-020-67482-9

**Published:** 2020-06-30

**Authors:** Yifan Liu, Ming Zhang, Yanju Shan, Gaige Ji, Xiaojun Ju, Yunjie Tu, Zhongwei Sheng, Jingfang Xie, Jianmin Zou, Jingting Shu

**Affiliations:** 1 Key Laboratory for Poultry Genetics and Breeding of Jiangsu Province, Poultry Institute, Chinese Academy of Agricultural Sciences, Yangzhou, 225125 Jiangsu China; 2 Jiangxi Academy of Agricultural Science, Nanchang, 330200 Jiangxi China

**Keywords:** Developmental biology, Gene expression analysis, Sequencing

## Abstract

Skeletal muscle fibers are primarily categorized into oxidative and glycolytic fibers, and the ratios of different myofiber types are important factors in determining livestock meat quality. However, the molecular mechanism for determining muscle fiber types in chickens was hardly understood. In this study, we used RNA sequencing to systematically compare mRNA and microRNA transcriptomes of the oxidative muscle *sartorius* (SART) and glycolytic muscle *pectoralis major* (PMM) of Chinese Qingyuan partridge chickens. Among the 44,705 identified mRNAs in the two types of muscles, 3,457 exhibited significantly different expression patterns, including 2,364 up-regulated and 1,093 down-regulated mRNAs in the SART. A total of 698 chicken miRNAs were identified, including 189 novel miRNAs, among which 67 differentially expressed miRNAs containing 42 up-regulated and 25 down-regulated miRNAs in the SART were identified. Furthermore, function enrichment showed that the differentially expressed mRNAs and miRNAs were involved in energy metabolism, muscle contraction, and calcium, peroxisome proliferator-activated receptor (PPAR), insulin and adipocytokine signaling. Using miRNA-mRNA integrated analysis, we identified several candidate miRNA-gene pairs that might affect muscle fiber performance, viz, gga-miR-499-5p/*SOX6* and gga-miR-196-5p/*CALM1*, which were supported by target validation using the dual-luciferase reporter system. This study revealed a mass of candidate genes and miRNAs involved in muscle fiber type determination, which might help understand the molecular mechanism underlying meat quality traits in chickens.

## Introduction

Improving meat quality has long been a goal of broiler breeding programs, especially for Chinese native breeds^1,2^. However, meat quality is difficult to define because it is a complex trait influenced by numerous factors^3^. As the main tissue determining meat quality, skeletal muscle is a heterogeneous tissue composed of different types of muscle fibers, varying in their biochemical and structural characteristics. Previous studies have found that different types of muscle fibers can influence meat quality traits, including meat color, tenderness, water-holding capacity, juiciness, and flavor^4,5^. In chickens, myofiber can be divided into red and white fibers, which are referred to as oxidative (type I and IIA) and glycolytic fibers (type IIB), respectively. Oxidative fibers exhibit slow contractility and oxidative metabolism based on mitochondrial oxidative phosphorylation, whereas glycolytic fibers have fast contractility and glycolytic metabolism^6,7^. Although the differences between various muscle fiber types in physiology and functionality have been well studied, the molecular regulation of their specification and maintenance in chickens remains largely unknown^8,9^.

miRNAs are highly conserved non-coding small RNAs that regulate gene expression at the post-transcriptional level in most biological processes. Emerging evidence has demonstrated that miRNAs are involved in skeletal muscle differentiation and development^10–12^. Recent studies have shown that some muscle-specific miRNAs play an important role in the regulation of muscle fiber type specification and maintenance in certain vertebrate species^13,14^. For example, miR-499 has been reported to control muscle fiber composition by repressing transcriptional repressors of slow-switch contractile protein genes, such as *SOX6* and *FNIP1*^15,16^. microRNA-139-5p in mice has been identified to suppress the expression of myosin heavy chains I and IIa via inhibition of the calcineurin(CaN)/NFAT signaling pathway^17^. Until now, the knowledge of miRNAs in regulating the chicken muscle fiber phenotype remains limited. According to the only report from Ma et al., miR-1611 in chickens can mediate the expression of *Six1*, thereby affecting the proliferation and differentiation of myoblasts and transformation of muscle fiber types^18^.

Qingyuan partridge chicken, an important indigenous breed in China, is popular because of its good meat quality^19^. High oxidative metabolism of this breed leads to desirable muscle characteristics, such as a high red muscle ratio and appealing meat color and flavor. In the present study, we compared the miRNA and mRNA differences between *pectoralis major* muscle (PMM) of the glycolytic type and *sartorius* muscle (SART) of the oxidative type and identify the key miRNA-mediated mechanism for muscle fiber type regulation. This work would contribute to the understanding of chicken meat quality control and improvement.

## Results

### Overview of RNA sequencing

We used Illumina deep sequencing to analyze the protein-coding transcript abundance in eight libraries based on the PMM and SART extracted from four Qingyuan partridge hens. After lower-quality reads were filtered, a total of 676.3 million clean reads were generated, more than 79.3% of which were mapped to the reference genome (Table S1). The GC contents of the clean data ranged from 46.7 to 49.0%, and the clean read quality scores of Q20 and Q30 were above 98.6% and 95.2%, respectively, demonstrating that the reliability and quality of the sequencing data were adequate for further analysis. The PCA analysis showed all eight samples were found to form two distinct clusters which were consistent with their groups’ descriptions (Figure S1).

Using *Galgal* 6.0 as the reference genome, 35,873 known transcripts and 8,832 novel protein-coding transcripts were identified in the data (Table S2). After the transcript expression levels were quantified, the average expression of the novel mRNAs (5.26) approximated to half of the known mRNAs (10.24). A total of 36,004 mRNAs were co-expressed in both the PMM and SART, while 3,958 and 4,743 transcripts were expressed only in the PMM and SART, respectively. All protein-coding transcripts corresponded to 15,962 genes, with an average of 2.8 transcripts per gene locus. Novel transcripts were identified in 37.6% of protein-coding genes in the present study (Table S2).

### Analysis of differentially expressed mRNAs

**Figure 1:**
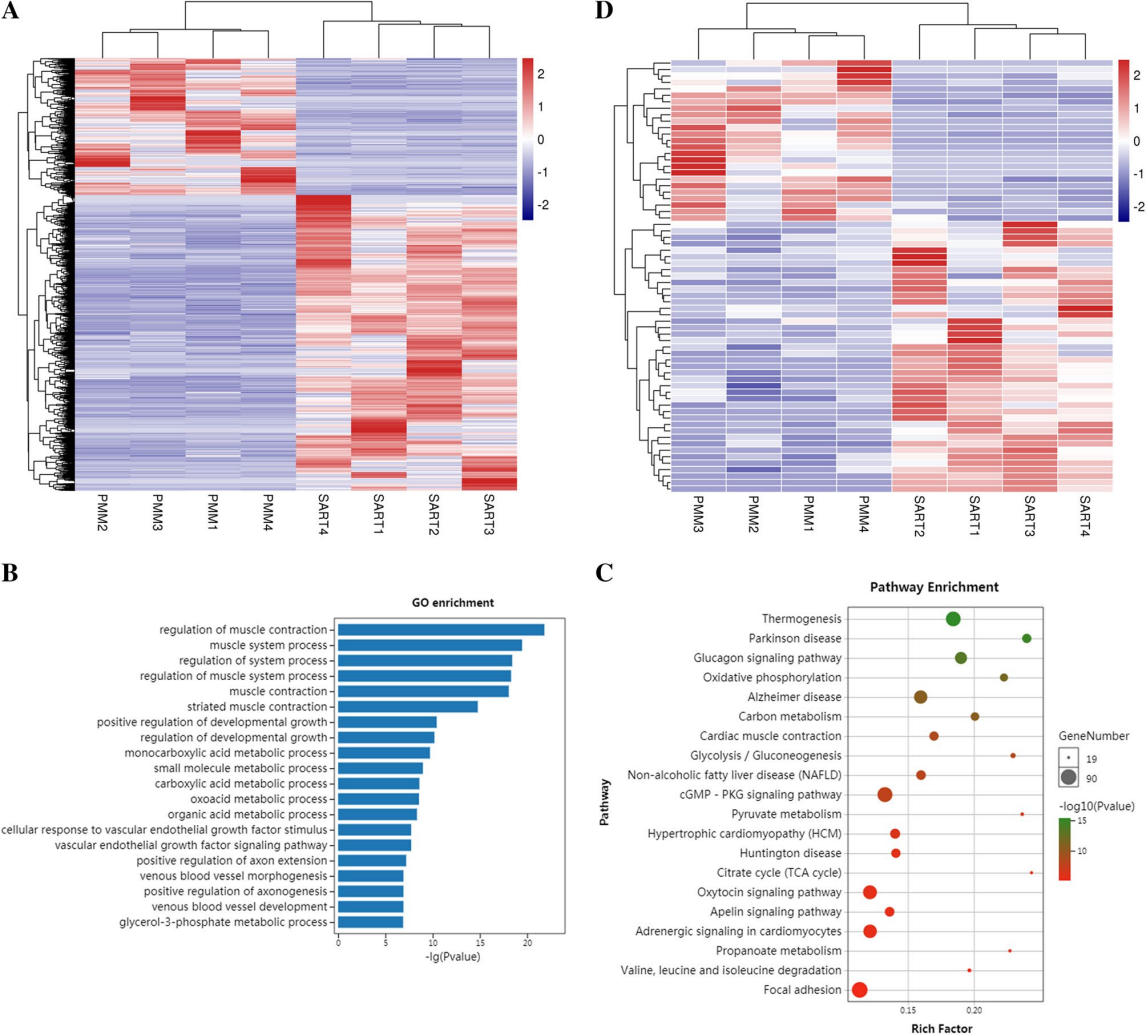
Gene and miRNA profiles between the *pectoralis major* muscle (PMM) and *sartorius* muscle (SART). (**A**) The hierarchical cluster analysis of differentially expressed mRNAs. (**B**) Top 20 significantly enriched GO terms of differentially expressed mRNAs in biological processes. (**C**) Top 20 significantly enriched KEGG pathways of differentially expressed mRNAs. The size and color of each bubble represent the amount of differentially expressed mRNAs enriched in the pathway and enrichment significance, respectively. (**D**) The hierarchical cluster analysis of differentially expressed miRNAs.

The analysis of differentially expressed genes in transcript level (DEGs) revealed a significant difference in multiple muscle tissues in chickens. There were 3,457 significant DEGs, including 2,364 up-regulated and 1,093 down-regulated mRNAs in the SART when compared to PMM (Table S3 & Fig. 1A). To further elucidate the functional roles of DEGs, we performed Gene Ontology (GO) and Kyoto Encyclopedia of Genes and Genomes (KEGG) pathway enrichment analysis for the DEGs. In biological processes, the enriched GO terms were associated with energy metabolism, blood circulation, muscle development, and contraction processes (Fig. 1B). The pathway enrichment revealed DEGs involved in several energy metabolism-related pathways, including oxidative phosphorylation, carbon metabolism and glycolysis/gluconeogenesis (Fig. 1B). Furthermore, the oxidative phosphorylation pathway was more often associated with up-regulated genes in SART, while glycolysis/gluconeogenesis was only enriched in PMM (Figure S2).

We also identified cardiac muscle contraction, adrenergic signaling in cardiomyocytes, focal adhesion, and the cGMP-PKG signaling pathway. Besides, most genes of CaN/NFAT signaling pathway, a core element of cGMP-PKG signaling pathway, were identified as DEGs in the present study.

### Overview of small-RNA sequencing

**Figure 2:**
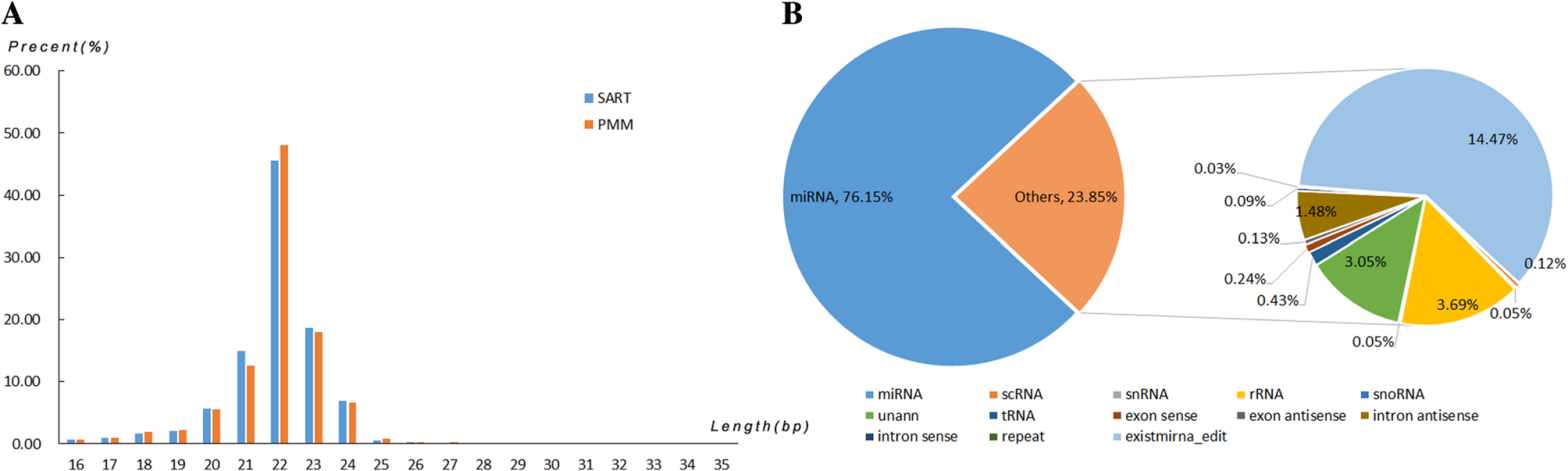
Overview of small RNA sequencing in chicken muscles. (**A**) The size distribution of all clean reads. (**B**) The distribution of raw reads mapped to the chicken genome. The percent of miRNA is approximately 76%, and the other 24% included rRNA, scRNA, snRNA, tRNA, snoRNA, repeat, exon sense/antisense, and intron sense/antisense.

A total of 102.5 Mb clean reads were obtained from eight libraries, representing a high ratio (> 90.8%) of clean reads (Table S4). After alignment with known small RNAs from Rfam, GenBank, and the reference genome, 76.1% of clean reads were identified as mature miRNAs, and the remaining small RNAs included rRNA, tRNA, scRNA, snRNA, snoRNA as well as exist-miRNA-edit (Fig. 2A). The size distribution of the reads was not significantly different in the PMM and SART libraries, and the majority of the reads had the lengths of 21–24 nt (Fig. 2B).

Known chicken miRNAs that were identified were 509, including 405 co-expressed in the PMM and SART libraries and 104 expressed in only one library (Table S5). Ten mature miRNAs with the highest expression comprised approximately 75% of all known miRNAs’ reads, showing a relatively abundant distribution. Specially, three miRNAs, viz. gga-miR-1a-3p, gga-miR-26a-2-5p and gga-miR-26a-5p, had more than 10,000,000 reads. The number of novel miRNAs predicted in this study reached 189 (Table S5). The sequencing frequencies of the novel miRNAs were much lower as compared to those of the known miRNAs.

### Analysis of differentially expressed miRNAs

A total of 67 differentially expressed miRNAs (DEMs) were identified, including 49 known and 18 novel miRNAs (Table S6 and Fig. 1D). From these, 42 miRNAs were up-regulated and 25 were down-regulated in SART. Among these DEMs, several related to muscle function were identified in this study, such as gga-miR-499, gga-miR-221, gga-miR-34a, and gga-miR-126. After target gene prediction, 5,862 target genes for the 67 DEMs were identified using a combination of three different tools. Among them, 1,537 mRNAs were found to be DEGs, which were assigned as intersection genes (Table S7). GO and KEGG pathway enrichment analysis of the intersection genes was performed to reveal the potential functions of the DEMs. The enriched GO terms mainly involved metabolic processes, cardiocyte differentiation and organ development biological processes (Figure S3A). The enriched pathways including the cGMP-PKG signaling pathway, adipocytokine signaling pathway, glycolysis/gluconeogenesis, insulin signaling pathway, AMPK signaling pathway, calcium signaling pathway and HIF-1 signaling pathway (Figure S3B).

### Validation of differentially expressed mRNAs and miRNAs by qRT-PCR

**Figure 3:**
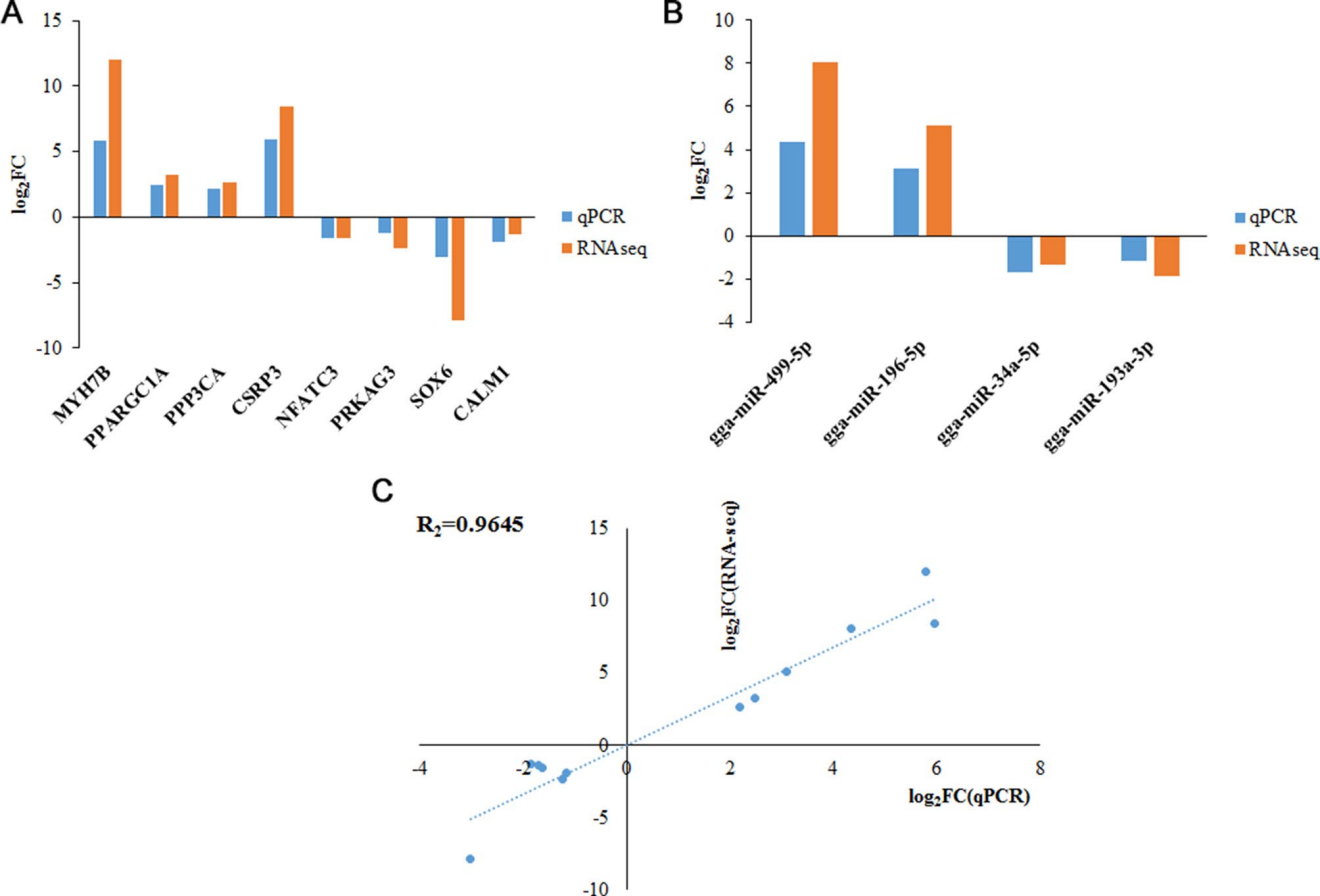
Validation of the differentially expressed mRNAs and miRNAs between the *pectoralis major* muscle (PMM) and *sartorius* muscle (SART). (**A**) Illustrating of qPCR confirmation results for eight selected differentially expressed mRNAs. (**B**) Illustrating of qPCR confirmation results for four selected differentially expressed miRNAs. (**C**) Regression analysis of log2(foldchange) values between RNA-seq and qPCR.

To validate the differential expression results of mRNAs and miRNAs, the relative expression of eight mRNAs (*MYH7B*, *NFATc3*, *PRKAG3*, *PPARGC1A*, *PPP3CA*, *CSRP3*, *SOX6,* and *CALM1*) and four miRNAs (gga-miR-499-5p, gga-miR-196-5p, gga-miR-34a-5p and gga-miR-193a-3p) was quantified by qRT-PCR. In Fig. 3A, B, all selected DEGs and DEMs showed the concordant expression patterns between RNA-seq and qPCR results. Also, the computational and experimental fold changes in the present study showed a strong positive correlation with R^2^ = 0.9645 (Fig. 3C).

### Construction of the miRNA-mRNA interaction network

By comparison with previous muscle-specific genes in pig, fish, and chicken^8,20–23^, more than 50 genes exhibiting similar expression patterns were screened in the current RNA-seq study (Table S8). These genes were involved in many functions related to muscle contraction and cytoskeleton, transcription factor, energy metabolism, Ca^2+^ homeostasis signaling and transport protein. Among these overlapped genes, most of the genes related to the myofiber characteristics in chickens, such as *PPARGC1A*, *PRKAG3* and *TGFB2*, were also identified in the present study^24,25^. Besides, a list of important transcription factors including *SOX6* and *CSRP3* were found to be express in a fiber type-specific manner^26,27^.

**Figure 4:**
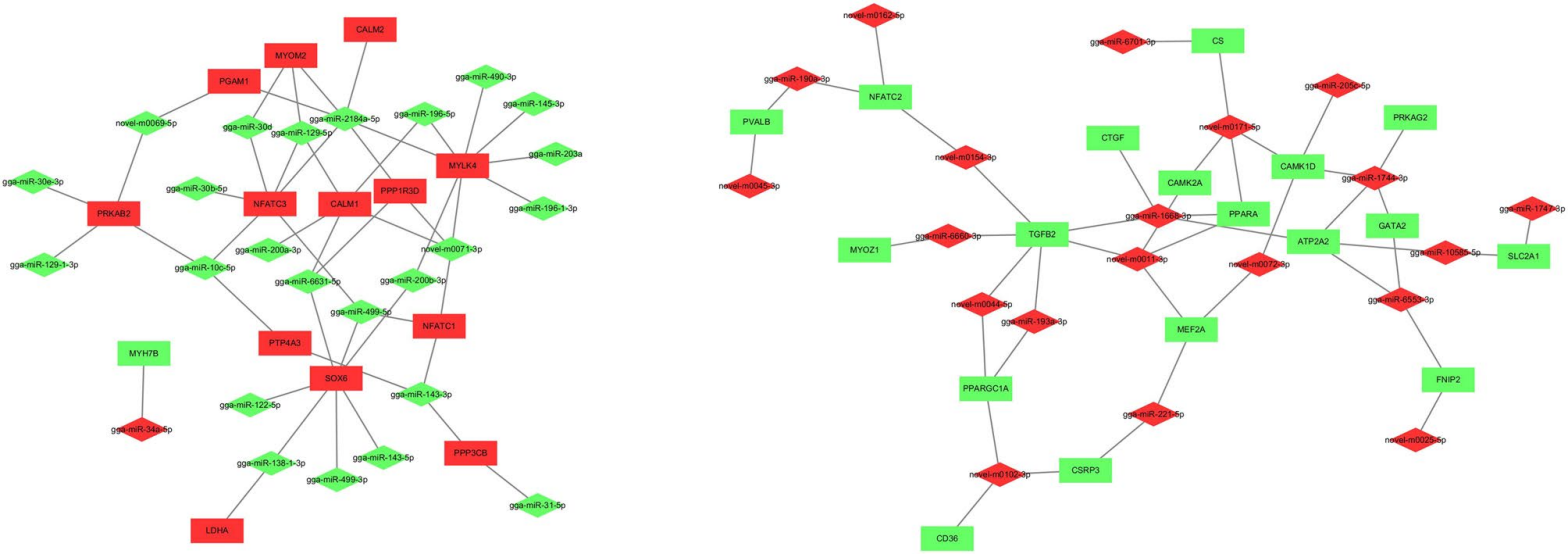
Interaction network of miRNAs and genes related to muscle fiber composition. The color of the node represents the expression regulation types of differentially expressed miRNAs and genes. Green means up-regulation and red means down-regulation in the SART.

**Figure 5:**
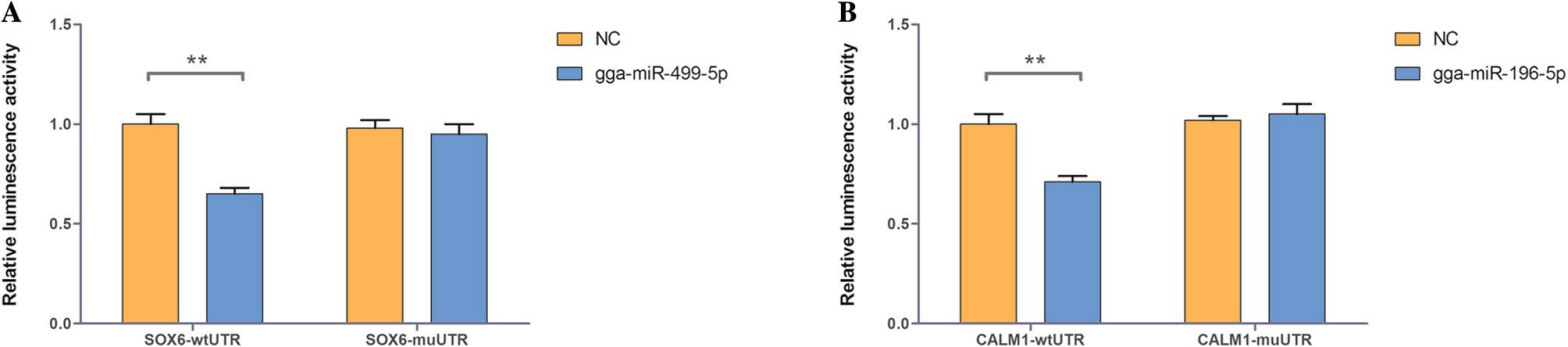
Validation of the predicted miRNA-target interaction with the 3′UTR luciferase reporter system. (**A**) Luciferase assays were performed on DF-1 cells co-transfected with the *SOX6* 3′UTR-WT and gga-miR-499-5p mimics, or *SOX6* 3′UTR-MUT and gga-miR-499-5p mimics. (**B**) Luciferase assays were performed on DF-1 cells co-transfected with the *CALM1* 3′UTR-WT and gga-miR-196-5p mimics, or *CALM1* 3′UTR-MUT and gga-miR-196-5p mimics. Data were expressed as means ± SD. The P value was calculated using the t-test. **P < 0.01.

To further understand and visualize the interactions of DEGs and DEMs related to muscle types, a miRNA-gene interaction network was constructed using the DEGs from Table S8 and all DEMs (Fig. 4). In the network, 92 interactions were identified. Two of the most significantly DEMs, gga-miR-196-5p and gga-miR-499-5p, were predicted to target *CALM1* and *SOX6*, respectively. A dual-luciferase reporter system was used to verify the binding relationship between the identified miRNAs and mRNAs. Luciferase assay showed that gga-miR-196-5p and gga-miR-499-5p could reduce the luciferase activity by binding to the sites on *CALM1* and *SOX6* 3′UTR, respectively (Fig. 5).

## Discussion

Muscle fiber type composition is one of the key factors that affect meat quality. For example, the high content of oxidative fiber contributes more to juiciness and flavor^28^. Muscle fiber characteristics are affected by muscle types, locations, and functions within an animal. Compared to the research progress in mammals, the molecular mechanism underlying the muscle fiber characteristics in chickens is largely unknown, especially in miRNA regulation. In this work, we used the RNA-seq technology to compare mRNAs and miRNAs in order to identify transcriptomic differences between PMM and SART, as well as typical oxidative and glycolytic muscle tissues in chickens^9,29^. To our knowledge, our work was the first exploring the mechanism of chicken muscle fiber type regulation based on skeletal muscles using the RNA-seq approach.

In the present study, a total of 3,457 mRNAs were found differentially expressed between PMM and SART using high-throughput sequencing. By comparing with earlier transcriptome results related to different muscles, over 50 DEGs overlapped between multiple studies, indicating their roles in skeletal myofiber regulation^8,20–23^. As expected, the expression levels of genes related to energy metabolism, slow-type muscle protein-encoding, muscle contraction and cytoskeleton were significantly higher in oxidative muscles than in glycolytic muscles, implying their roles in meat quality regulation. For example, *CSRP3* belongs to the cysteine and glycine-rich protein family, which play an important role in muscle fiber differentiation, is also highlighted as a fiber type-specific isoform in different species^27,30^. The mutation of another muscle-specific gene *PRKAG3* has an influence on muscle metabolism and fiber type in pig, and have been shown to have a strong association with meat quality^31,32^. Similar to previous studies, the functional enrichment analysis demonstrated that these DEGs were involved in energy metabolism, muscle contraction, calcium homeostasis, peroxisome proliferator-activated receptor (PPAR), insulin and adipocytokine signaling. The newly identified DEGs might provide valuable information for the regulation of the chicken skeletal muscle phenotype.

Several groups have investigated miRNA expression between oxidative and glycolytic skeletal muscles in mammals and fish^14,33–36^. In this study, we have identified 899 chicken miRNAs in the two types of chicken muscles. Similar to other studies, most miRNA reads came from a few miRNAs^37,38^. Different from previous reports, the miRNAs with the highest expression were not differentially expressed between muscles. We detected 46 DEMs, many of which were related to muscle functions, including myofiber type switching. For example, as one of the most up-regulated miRNAs in the SART, miR-499 was shown to regulate the slow-twitch phenotype by targeting NFATc1/MEF2C and Fnip1/AMPK circuits^15,39^. Gga-miR-143 was another important miRNA. In swine, overexpression of miR-143 could induce the increase of slow fibers through targeting the *HDAC4* expression^40^. Also, we found some DEMs identified here that involving muscle cell differentiation and energy metabolisms, such as gga-miR-221, gga-miR-34a and gga-miR-126^41–43^. Moreover, the biological functions of a few miRNAs in chicken muscles was revealed through the GO and pathway analysis of intersection genes. Our results provide novel information regarding the regulatory roles of these miRNAs.

To further understand the mechanism how miRNAs and their targets regulated muscle fiber types, an interaction network between miRNAs and mRNAs related to muscle fiber composition was constructed. One of the core genes in the network, *SOX6* was targeted by seven miRNAs including gga-miR-499-5p and gga-miR-499-3p. As an important member of the SRY-related high mobility group (HMG) box (*SOX*) family of the transcription factors, *SOX6* was highly expressed in skeletal muscle and acted as a repressor of fetal slow-twitch-specific gene expression in zebrafish and mice^44,45^. Further studies showed that *SOX6* was a target of miR-499-5p^16,46^. As the top up-regulated miRNA in the SART, miR-196-5p was predicted to suppress *CALM1* and *MYLK4* expression. Based on KEGG enrichment analysis, *CALM1* and *MYLK4* were key elements of cGMP–PKG and calcium signaling pathways, which were vital in the regulation of muscle fiber type transformation^47,48^. In our previous work, *PPARGC1A* was reported being essential to slow muscle fiber formation in chicken myoblast cells, and its polymorphisms were associated with skeletal myofiber type traits^8,24^. We found that gga-miR-193-3p inhibited *PPARGC1A* in chicken muscles. Interestingly, our data on target validation provided strong evidence that *SOX6* was a target of gga-miR-499-5p, whereas *CALM1* was a target of gga-miR-196-5p. We therefore hypothesized that these miRNAs could target sequences in these genes to regulate the chicken muscle fiber phenotype.The CaN/NFAT signaling pathway provides an important link to calcium pattern dependent signaling and is implicated in the control of skeletal muscle fiber gene expression^49,50^. In the present study, several DEGs including *CALM1*, *CALM2*, *PPP3CA*, *NFATc1* and *NFATc3,* were implicated in the CaN/NFAT signaling pathway, which was consistent with the previous studies^8^. Based on the miRNA-mRNA interaction network, various miRNAs were predicted to target these genes. Among these identified miRNAs, gga-miR-143-5p, gga-miR-499-5p and gga-miR-129-3p had multiple target genes, which were implicated in CaN/NFAT signaling, suggesting their roles in chicken muscle fiber regulation through the CaN/NFAT signaling pathway.

In summary, we characterized mRNA and miRNA transcript profiles of the PMM and SART muscles by RNA sequencing. We identified and characterized the DEGs and DEMs that were involved in chicken muscle fiber regulation. By analyzing these differentially expressed mRNAs and miRNAs, a miRNA-mRNA network associated with muscle fiber type composition was established. This study expanded our understanding of molecular mechanisms underlying meat quality traits in chickens.

## Materials and methods

### Ethics statement

All animal experiments were approved by the Animal Care and Use Committee at the Poultry Institute, Chinese Academy of Agricultural Science (Approval ID: S20180605). All these experiments followed relevant guidelines and regulations set by the Ministry of Agriculture and Rural Affairs of the People's Republic of China.

### Animal, muscle sampling and RNA extraction

A total of 200 Qingyuan partridge chickens were obtained from Tinoo’s Foods Co., Ltd (Guangdong, China). All experimental birds were lived under the same environment and were raised using a standardized feeding method with free access to water. At the 140th day of age indicating sexual maturity, four female chickens with similar weights (1,428–1477 g, 1455 g averagely) were selected for transcriptome analysis. As previously described^7^, skeletal muscle samples were obtained from the intermediate section of the PMM and SART immediately after exsanguination, rapidly frozen in liquid nitrogen, and then stored at − 80 °C for further use.

Total RNAs were extracted from the PMM and SART muscle samples of the four hens using TRIzol reagent (Invitrogen, CA, USA) according to the manufacturer’s instructions. The RNA quality was checked using NanoDrop 2000 spectrophotometer (Thermo, USA) and Agilent 2100 Bioanalyzer (Agilent Technologies, CA, USA), respectively. RNA integrity number (RIN) ≥ 7 was set as the cutoff for RNA quality.

### RNA sequencing and mRNA analysis

A total of eight cDNA libraries were constructed with four PMM and four SART muscle tissues, and 3 μg total RNA per sample was used as the input material for a cDNA library. After total RNAs were extracted, rRNAs were removed, and then the enriched RNAs were fragmented into short fragments and reverse transcribed into cDNAs. Double-stranded cDNAs were synthesized by replacing dTTPs with dUTPs in the reaction buffer used in second-strand cDNA synthesis. The resulting double-stranded cDNAs were ligated to adaptors after being end-repaired and A-tailed. Then, uracil-N-glycosylase (UNG) was used to digest the second-strand cDNAs. The digested products were size selected by agarose gel electrophoresis, PCR amplified and sequenced by Gene Denovo Biotechnology Co. (Guangzhou, China) using Illumina HiSeq 4000.

Raw data in FASTQ format were first processed with in-house scripts. The sequencing raw reads were obtained and used in further analysis after removing reads containing adapters, reads containing ploy-N, and low-quality reads from raw data. The clean reads of each sample were then mapped to the reference genome (*Galgal* 6.0) by Tophat2 (version 2.1.1)^51^. Known and novel transcripts from the TopHat alignment results were constructed and identified by the Reference Annotation Based Transcript (RABT) assembly of Cufflinks v2.1.1^52^. Transcript abundance was quantified by the RSEM software^53^. The transcript expression levels were normalized by using the Fragments Per Kilobase of transcript per Million mapped reads (FPKM) method. mRNAs with a P value < 0.05 and fold change ≥ 2 were then identified as significant DEGs using the edgeR package^54^.

### Small RNA sequencing and miRNA analysis

Eight small RNA libraries were also constructed using the same RNA samples as those used for RNA sequencing. After extracting total RNAs from muscle tissues, low molecular weight RNAs were separated by polyacrylamide gel electrophoresis (PAGE). Then, the 3′ adapters and 5′ adapters were ligated to the RNAs as well. The ligation products were reverse transcribed by PCR amplification, and the 140–160 bp PCR products were enriched to generate a cDNA library and sequenced using Illumina HiSeq 2500 by Gene Denovo Biotechnology Co., Ltd (Guangzhou, China).

After quality control processes, the clean reads were mapped to the chicken genome *Galgal* 6.0 using Bowtie2^55^ and classified by aligning them against the GeneBank (Release 209.0), Rfam (11.0) and miRBase (21.0) databases as previously described^37^. Sequences matching Gallus gallus miRBase were considered known miRNAs. According to their genome positions and hairpin structures predicted by the software Mireap (v0.2), novel miRNA candidates were identified^56^. The miRNA expression level was calculated and normalized to transcripts per million (TPM). The criteria of fold change ≥ 1.5 and a P-value < 0.05 was used to screen the differentially expressed miRNAs (DEMs). The RNAhybrid (v2.1.2) + SVM_LIGHT (v6.01), Miranda (v3.3a) and TargetScan (version 7.0) software were used to predict targets of miRNAs^57^.

### Function enrichment analysis

All DEGs and target genes of DEMs were annotated and classified by Gene Ontology (GO) and Kyoto Encyclopedia of Genes and Genomes (KEGG) pathway analysis with the OmicShare tools (https://www.omicshare.com/tools/). The results with P value < 0.05 were considered significantly enriched.

### Validation by real-time quantitative PCR

To validate the differential expression result from sequencing, eight mRNAs and four miRNAs were selected for qRT-PCR. The total RNAs for sequencing were reverse transcribed into cDNAs by the PrimeScript RT reagent kit (TaKaRa, Dalian, China). Then, qRT-PCR was conducted using KAPA SYBR Fast universal qPCR kit (Kapa Biosystems, USA). Glyceraldehyde-3-phosphate dehydrogenase (GAPDH) genes were used as the internal reference. All primers are shown in Table S9. For miRNA expression, 3 µg of miRNA was subject to reverse transcription with the miRNA 1st-Strand cDNA Synthesis Kit (Vazyme, Nanjing, China). qRT-PCR was then performed by miRNA Universal SYBR qPCR Master Mix (Vazyme, Nanjing, China). The U6 snRNA was used as the internal reference. All qRT-PCR reaction was carried out in the ABI 7,500 Real-Time PCR system (Applied Biosystem, CA, USA) with triplicate reactions for each sample. The relative expression levels of the genes and miRNAs were quantified using the 2(− ΔΔCt) method^58^. The independent sample T-test procedure of SPSS (Version 20.0) was used to assess expression differences between PMM and SART muscle samples.

### Luciferase reporter assay

Luciferase reporter experiments were performed on DF-1 cells. The gga-miR-499-5p and gga-miR-196-5p mimics and the negative control mimic were purchased from Ribobio Co., Ltd (Guangzhou, China). Wild-type (WT) and mutant (MUT) luciferase reporter vectors were synthesized based on the PmiR-RB-Report vector by the same company (Table S9). Cells were transfected using either wild-type or mutant constructs, with the specific mimics or negative control mimic. And 48 h later, Dual-Glo Luciferase Assay System (E2920, Promega, MA, USA) was utilized to detect the luminescence.

## Supplementary information

Supplementary figures

Supplementary table S1

Supplementary table S2

Supplementary table S3

Supplementary table S4

Supplementary table S5

Supplementary table S6

Supplementary table S7

Supplementary table S8

Supplementary table S9

## Data Availability

The data sets supporting the results presented here are available in the Sequence Read Archive (SRA) repository under accession number PRJNA578179.
