# Supplementary material for: miRNA–mRNA network regulation in the skeletal muscle fiber phenotype of chickens revealed by integrated analysis of miRNAome and transcriptome
Source: Sci Rep. 2020 Jun 30;10:10619. doi: 10.1038/s41598-020-67482-9 (PMC7326969; doi:10.1038/s41598-020-67482-9)
Supplement: Supplementary file 1 — Supplementary figures [file 41598_2020_67482_MOESM1_ESM.docx]

**miRNA-mRNA network regulation in the skeletal muscle fiber phenotype of chickens revealed by integrated analysis of miRNAome and transcriptome**

Yifan Liu^1§^, Ming Zhang^1§^, Yanju Shan^1^, Gaige Ji^1^, Xiaojun Ju^1^, Yunjie Tu^1^, Zhongwei Sheng^1^, Jingfang Xie^2^, Jianmin Zou^1^, and Jingting Shu^1^*

^1^Key Laboratory for Poultry Genetics and Breeding of Jiangsu Province, Poultry Institute, Chinese Academy of Agricultural Sciences, Yangzhou 225125, Jiangsu, China.

^2^Jiangxi Academy of Agricultural Science, Nanchang 330200, Jiangxi, China

^§^These authors contributed equally to this work

Correspondence and requests for materials should be addressed to J.S. (email: shujingting@163.com)


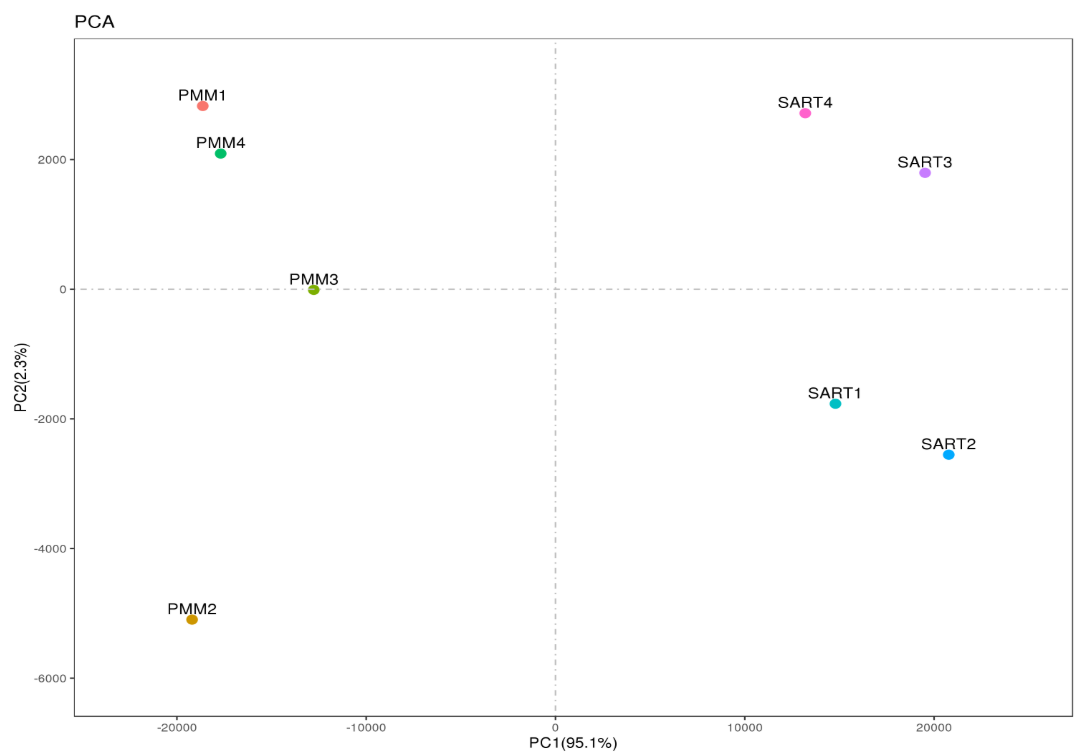
 **Figure S1:** Principal components plot of eight samples used in RNA sequencing.


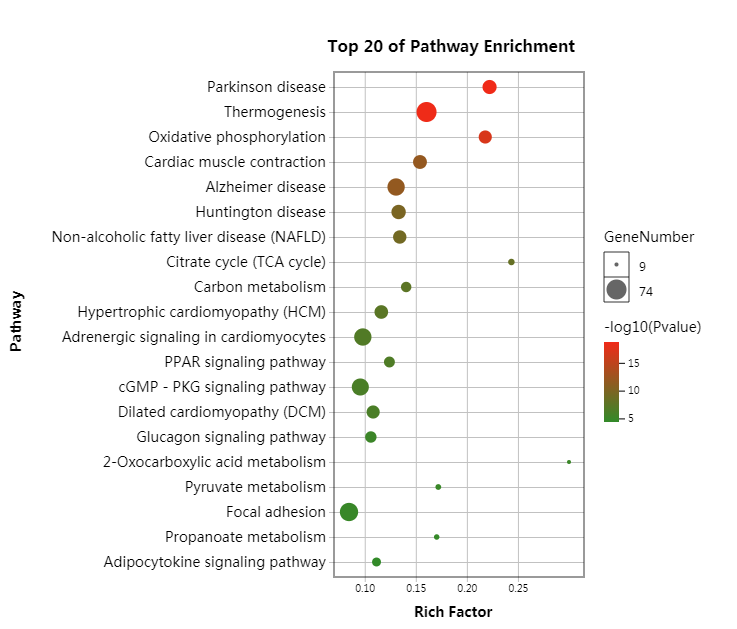


**A**


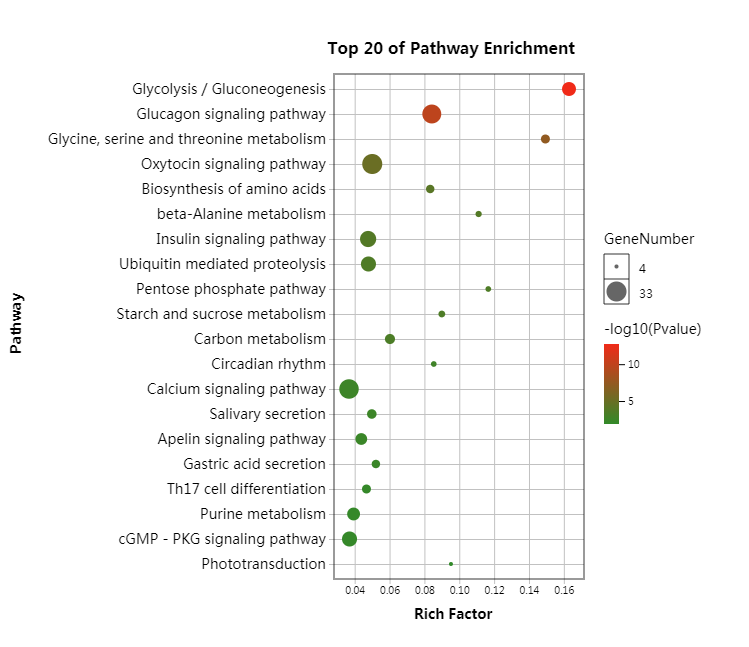


**B**

**Figure S2:** Top 20 significantly enriched KEEG pathways of up-regulated (A) and down-regulated (B) mRNAs in SART.
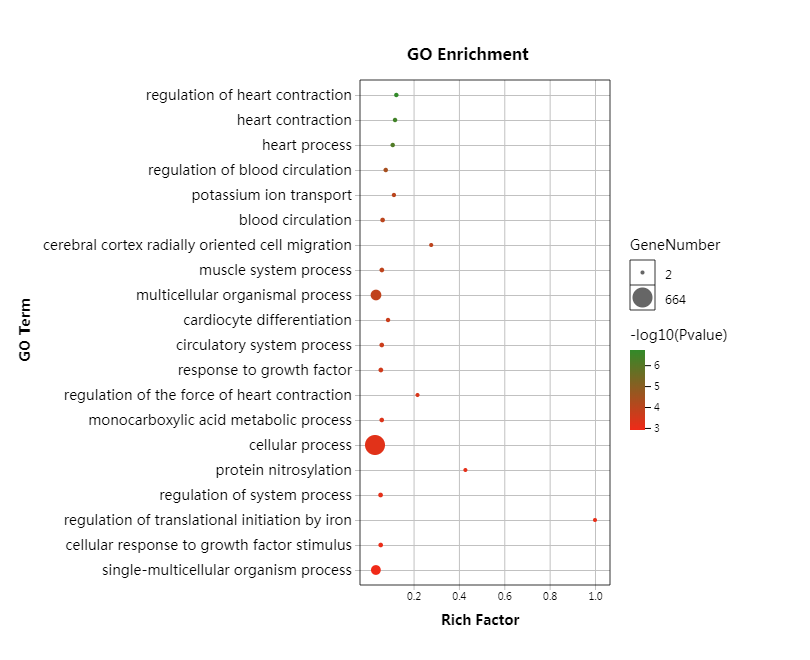
**A**


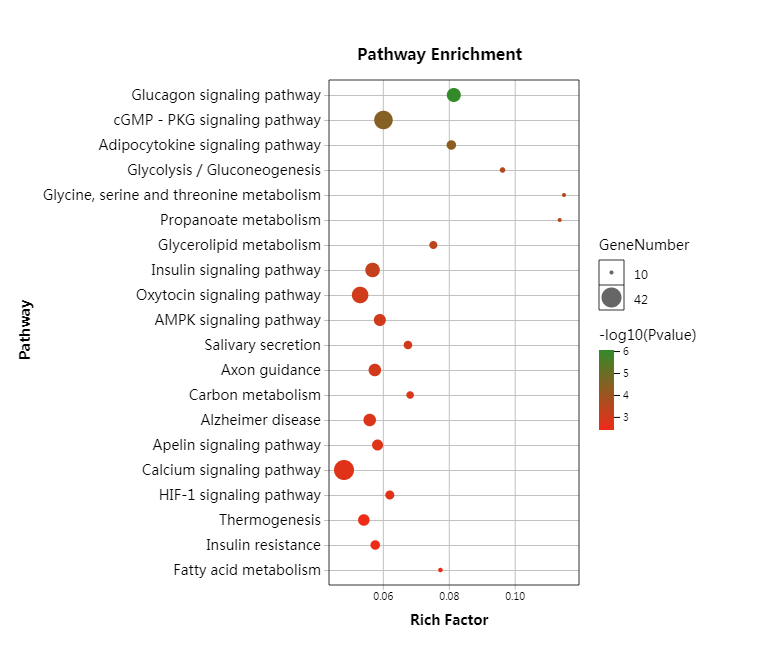


**B**

**Figure S3:** Top 20 significantly enriched GO terms (A) and KEEG pathways (B) of target genes differentially expressed miRNAs.
